# High-performance dialyzers and mortality in maintenance hemodialysis patients

**DOI:** 10.1038/s41598-021-91751-w

**Published:** 2021-06-10

**Authors:** Masanori Abe, Ikuto Masakane, Atsushi Wada, Shigeru Nakai, Eiichiro Kanda, Kosaku Nitta, Hidetomo Nakamoto

**Affiliations:** 1 The Committee of Renal Data Registry, The Japanese Society for Dialysis Therapy, Tokyo, Japan; 2 Division of Nephrology, Hypertension and Endocrinology, Department of Internal Medicine, Nihon University School of Medicine, Tokyo, Japan; 3 Yabuki Hospital, Yamagata, Japan; 4 Department of Nephrology, Kitasaito Hospital, Asahikawa, Japan; 5 Department of Clinical Engineering, Fujita Health University, Aichi, Japan; 6 Medical Science, Kawasaki Medical School, Okayama, Japan; 7 Department of Nephrology, Tokyo Women’s Medical University, Tokyo, Japan; 8 Department of General Internal Medicine, Saitama Medical University, Saitama, Japan

**Keywords:** Nephrology, Renal replacement therapy, Haemodialysis

## Abstract

Few data are available regarding the association of dialyzer type with prognosis. In Japan, dialyzers are classified as types I, II, III, IV, and V based on β_2_-microglobulin clearance rates of < 10, < 30, < 50, < 70, and ≥ 70 mL/min, respectively. We investigated the relationship of the 5 dialyzer types with 1-year mortality. This nationwide cohort study used data collected at the end of 2008 and 2009 by the Japanese Society for Dialysis Therapy Renal Data Registry. We enrolled 203,008 patients on maintenance hemodialysis who underwent hemodialysis for at least 1 year and were managed with any of the 5 dialyzer types. To evaluate the association of dialyzer type with 1-year all-cause mortality, Cox proportional hazards models and propensity score-matched analyses were performed. After adjustment of the data with clinicodemographic factors, the type I, II, and III groups showed significantly higher hazard ratios (HRs) than the type IV dialyzers (reference). After adjustment for Kt/V and β_2_-microglobulin levels, the HRs were significantly higher in the type I and II groups. After further adjustment for nutrition- and inflammation-related factors, the HRs were not significantly different between the type IV and type I and II groups. However, type V dialyzers consistently showed a significantly lower HR. With propensity score matching, the HR for the type V dialyzer group was significantly lower than that for the type IV dialyzer group. Additional long-term trials are required to determine whether type V dialyzers, which are high-performance dialyzers, can improve prognosis.

## Introduction

The number of patients receiving hemodialysis is increasing worldwide^1^. Dialyzer technology is moving to high permeability and high biocompatibility because the use of such dialyzers may improve mortality in patients on hemodialysis. Membrane dialyzers are classified as either low- or high-flux. High-flux membranes with high biocompatibility and large pores are recommended by the European Renal Best Practice guidelines to reduce morbidity and mortality^2^. Meanwhile, cellulose membranes with poor biocompatibility are discouraged in Kidney Disease Outcomes Quality Initiative guidelines^3^.

High-flux dialyzers have an ultrafiltration rate ≥ 15 mL/mmHg/h and β_2_-microglobulin (β2MG) clearance rate ≥ 15 mL/min^4^. On the other hand, dialyzers are classified in Japan as type I to type V by β2MG clearance rates of < 10, < 30, < 50, < 70, and ≥ 70 mL/min, respectively, at blood and dialysate flow rates of 200 mL/min and 500 mL/min, respectively^5^. Therefore, type II to V dialyzers are considered high-flux dialyzers in Japan, which means that the only low-flux dialyzers are type I dialyzers. Furthermore, type IV and V dialyzers in Japan include high-performance membrane (HPM) or super high-flux dialyzers. Over 90% of patients on hemodialysis in Japan are managed with HPM or super high-flux dialyzers^4,5^. However, few data are available on the effects of HPM or super high-flux dialyzers on prognosis. Accordingly, we performed a prospective cohort study of a national registry of patients on hemodialysis in Japan to ascertain the effects of dialyzer type on mortality.

## Methods

### Database

All data used in this study were obtained from the Japanese Society for Dialysis Therapy Renal Data Registry (JRDR). As previously described, JSDT volunteers surveyed dialysis patients in Japan in 2008 and 2009^6–8^. In the 2008 survey, data were obtained on 282,622 patients undergoing hemodialysis at 4072 facilities, whereas data were obtained in the 2009 survey on 290,675 patients at 4125 facilities^9,10^. This work, based on existing data, was performed with adherence to Japanese laws concerning privacy protection, the tenets of the Declaration of Helsinki, and the 2015 Ethical Guidelines for Medical and Health Research Involving Human Subjects by the Japanese Ministries of Education, Culture, Sports, Science and Technology and of Health, Labour and Welfare. The Medicine Ethics Committee of the Japanese Society for Dialysis Therapy approved the protocol of the study and waived the need for informed consent due to the use of de-identified data. The study was registered with the University Hospital Medical Information Network (UMIN000018641).

### Study design

A 1-year nationwide cohort study was performed retrospectively using data recorded in the JRDR up to December 31, 2008^9^, and December 31, 2009^10^. Data up to December 31, 2008, were considered the baseline data. Eligibility criteria were receipt of maintenance dialysis for at least 1 year by the end of the 2008 and the use of any of the 5 types of dialyzers (i.e., types I–V). We excluded patients who underwent dialysis for less than 2 h/day or fewer than 3 times a week, received an organ transplant, or underwent hemodiafiltration and peritoneal dialysis, patients younger than 18 years old, and patients with incomplete records concerning date of birth, dialysis initiation, dialyzer use, or outcome.

**Figure 1:**
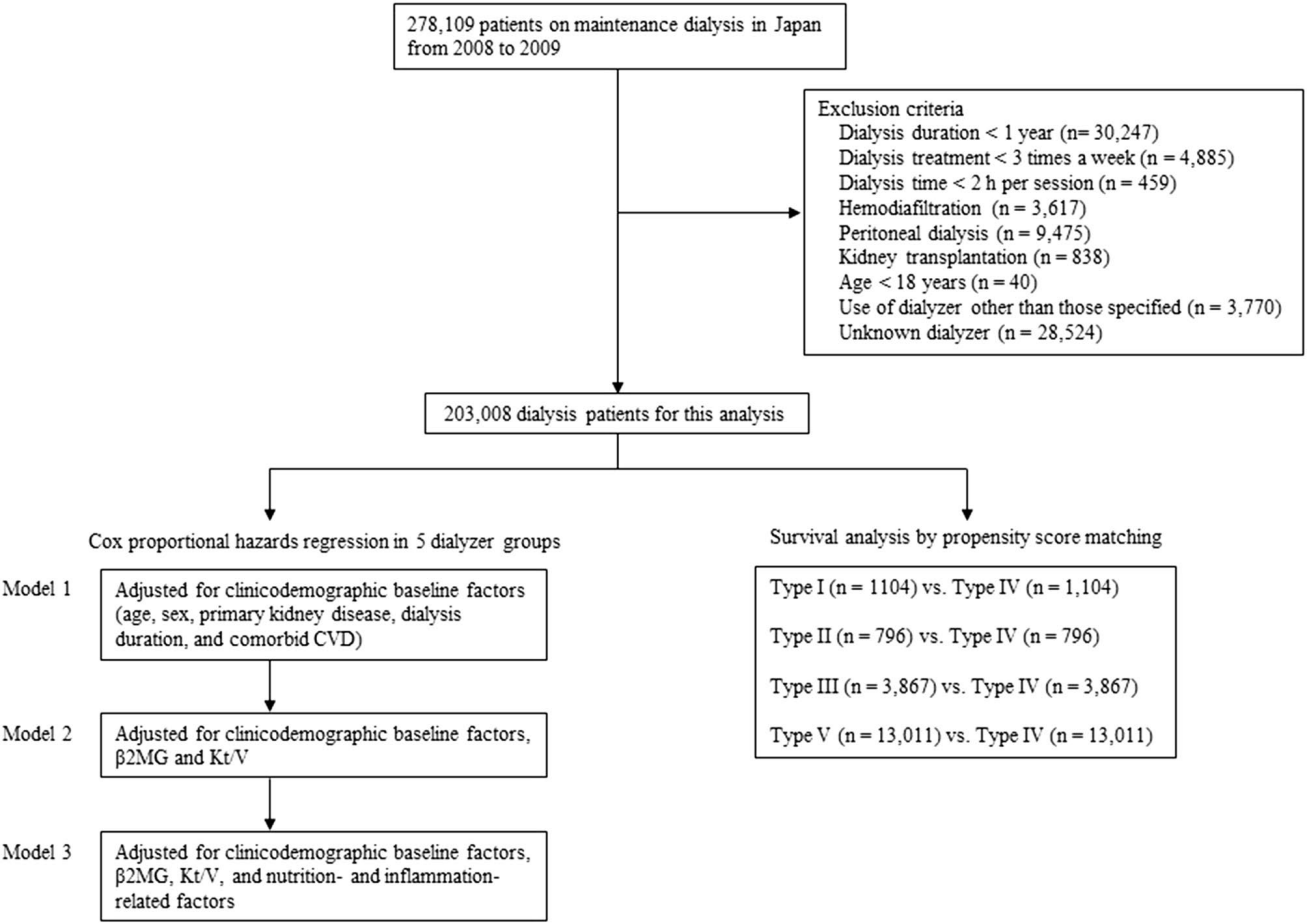
Flowchart of study participants. *β2MG *β2-microglobulin, *CVD* cardiovascular disease.

In total, 278,109 patients were registered in the database by December 31, 2008; 203,008 remained after exclusions (Fig. 1). Clinicodemographic and medical history data were gathered and included age, sex, height, postdialysis body weight, dialysis type and vintage, primary cause of the end-stage kidney disease, and history of vascular complication (e.g., cerebral infarction, cerebral hemorrhage, myocardial infarction, and limb amputation). Dates of death were extracted from the JRDR database at the end of 2009. All-cause mortality in the 1-year observation period was the primary outcome measure.

Blood was sampled in each dialysis center and assayed, generally less than 24 h after sampling. The most recent values at the time of the survey were recorded and included hemoglobin, serum albumin, phosphate, calcium, β2MG, C-reactive protein, normalized protein catabolic rate (nPCR), simplified creatinine index (SCI) and dialysis dose^11,12^. The dialysis dose was calculated using single-pool Kt/V for urea (Kt/V)^4,13^. SCI was calculated using the formula of Canaud et al.^14^.

### Statistical analysis

Data are expressed as proportions, with means ± standard deviation or median [interquartile range] as needed. The chi-square test was used for categorical variables, whereas the Student’s t test was used for continuous variables. Categorical data were compared between groups with repeated-measures ANOVA and Tukey’s honestly significant difference or Kruskal–Wallis test, as required.

Survival analyses were performed as reported previously^7^. Cox proportional hazards regression were used to determine whether clinicodemographic baseline factors (e.g., age, sex, primary kidney disease, dialysis vintage, and comorbid cardiovascular disease [CVD]) could predict survival at 1-year follow-up in Model 1. Patients were divided a priori into 7 groups according to dialysis vintage to investigate the dose–response relationship of dialysis vintage category with mortality. Further analyses were adjusted for β2MG and dialysis dose in Model 2. In addition, patients were divided a priori into 8 groups according to the single-pool Kt/V— < 0.8 and ≥ 2.0 at 0.2 increments—to investigate the dose–response relationship of Kt/V category with mortality. Further analyses were carried out with adjustment for factors related to nutrition and inflammation, which included body mass index (BMI), hemoglobin, serum C-reactive protein, and albumin concentrations, nPCR, and SCI in Model 3. Patients were additionally divided a priori into 6 groups to investigate the dose–response relationship of the following parameters with mortality according to the nPCR: < 0.5 and ≥ 1.3 g/kg/day at 0.2 g/kg/day increments; according to the serum albumin level: < 3.0 and ≥ 4.5 g/dL at 0.5 g/dL increments; and according to the BMI: < 16 and ≥ 28 kg/m^2^ at 2 kg/m^2^ increments. Age, β2MG, hemoglobin level, C-reactive protein, and SCI level were treated as continuous variables.

Survival analyses were performed using Cox proportional hazards regression to determine whether the type of dialyzer could predict survival at the 1-year follow-up. In the final analysis, we assessed the relationship between dialyzer type and all-cause mortality. Patients were divided into 5 groups according to the type of dialyzer used. The above-mentioned clinicodemographic factors, as well as dialysis dose and nutrition- and inflammation-related factors measured at baseline, were used to adjust the models. The type IV dialyzer group was considered the reference group because it is the most commonly used dialyzer in Japan^15^.

Finally, to limit the effects of potential confounding and treatment selection bias, significant baseline covariates were adjusted by propensity score matching. Propensity scores were calculated using factors found to contribute to mortality, including the above clinicodemographic factors, dialysis dose, and nutrition- and inflammation-related factors. These factors were assessed using univariate Cox proportional hazards regression analysis. The propensity score was then used to perform 1:1 matching of patients treated with the type IV dialyzer as reference with those treated with the other types of dialyzers, resulting in 1104, 796, 3867, and 13,011 matched pairs (I, II, III, and V, respectively). In addition, all-cause mortality was compared among the propensity score-matched patients. Furthermore, the scores were incorporated into statistical models using inverse probability of treatment weighting (IPTW) to compensate for potential confounding by indications for dialysis. The fit of these models was evaluated using the C-statistic for propensity scores.

The conventional method for multivariate regression was used to impute any missing covariate data. SAS software, version 14.2 (SAS Institute, Inc., Cary, NC) was used for all statistical analyses and significance was set at P < 0.05.

## Results

### Baseline characteristics

After exclusions, 203,008 hemodialysis patients were included in this analysis. The characteristics of the included patients are shown in Table 1 and can be summarized as follows: mean age, 65.3 ± 12.5 years; median dialysis vintage, 7 [4–12] years; female sex, 38.9%; BMI, 21.1 ± 3.5 kg/m^2^; CVD history (including coronary artery disease, ischemic or hemorrhagic stroke, and limb amputation), 24.9%; albumin, 3.7 ± 0.4 g/dL; and hemoglobin, 10.4 ± 1.2 g/L. Glomerulonephritis (43.0%) was the most common cause of end-stage kidney disease, followed by diabetic nephropathy (33.2%) and nephrosclerosis (7.1%). The proportions of categorical variables are shown in Supplementary Table 1. During the observation period, 15,900 deaths were recorded (6675 cardiovascular-related deaths, 2860 infection-related deaths, 1482 cancer-related deaths, and 4883 other deaths).

**Table 1:** Baseline clinicodemographic characteristics and laboratory values in 203,008 hemodialysis patients.

Variable	Value
Number of patients (% female)	203,008 (38.9)
Age (years)	65.3 ± 12.5
Dialysis vintage (years)	7 [4–12]
**CVD comorbidity (%)**	24.9
Coronary artery disease	7.3
Ischemic stroke	14.9
Hemorrhagic stroke	4.9
Limb amputation	3.0
**Primary kidney disease (%)**	
Glomerulonephritis	43.0
Diabetic nephropathy	33.2
Nephrosclerosis	7.1
Others	16.7
Smoking (%)	14.9
Body mass index (kg/m^2^)	21.1 ± 3.5
Hemoglobin (g/dL)	10.4 ± 1.2
Calcium (mg/dL)	9.0 ± 0.8
Phosphate (mg/dL)	5.3 ± 1.5
Intact PTH (pg/mL)	121 [61–206]
C-reactive protein (mg/dL)	0.12 [0.05–0.39]
β_2_- microglobulin (mg/L)	27.2 ± 6.9
Total cholesterol (mg/dL)	154 ± 35
HDL-cholesterol (mg/dL)	49 ± 16
Albumin (g/dL)	3.7 ± 0.4
Kt/V	1.41 ± 0.29
nPCR (g/kg/day)	0.88 ± 0.18
SCI (mg/kg/day)	21.0 ± 3.0

*CVD* cardiovascular disease, *HDL* high-density lipoprotein, *nPCR* normalized protein catabolic rate, *PTH* parathyroid hormone, *SCI* simplified creatinine index.

### All-cause mortality by clinicodemographic factors, dialysis dose, and nutrition- and inflammation-related factors

Table 2 shows the hazard ratios (HRs) for variables deemed potential predictive factors for mortality. Significant predictors of mortality were male sex, older age, dialysis vintage, and comorbid CVD. In addition, an end-stage kidney disease cause other than glomerulonephritis was a significant predictor of mortality. In contrast, a higher dialysis dose, assessed by single-pool Kt/V, and lower β2MG level were correlated with lower risk of mortality. Furthermore, poor nutritional status, shown by lower hemoglobin, serum albumin, BMI, nPCR, and %CGR, were linked to higher mortality. Increased inflammation, as determined by a higher C-reactive protein level, was also related to increased mortality in patients receiving hemodialysis.

**Table 2:** Hazard ratios and 95% confidence intervals for variables evaluated as potential predictors of mortality among all patients.

	HR	95% CI	P value
**Sex**			
Male	1.000	Reference	–
Female	0.919	0.892–0.964	< 0.0001
**Age (years)**			
1-year increase	1.064	1.059–1.062	< 0.0001
**Dialysis vintage (years)**			
> 1 to < 5	0.972	0.937–1.008	0.128
≥ 5 to < 10	1.000	Reference	–
≥ 10 to < 15	0.884	0.845–0.033	< 0.0001
≥ 15 to < 25	0.872	0.831–0.917	< 0.0001
≥ 25 to < 30	0.728	0.681–0.781	< 0.0001
≥ 30	0.873	0.769–0.990	0.031
**Primary kidney disease**			
Glomerulonephritis	1.000	Reference	–
Diabetic nephropathy	1.551	1.499–1.603	< 0.0001
Nephrosclerosis	1.584	1.502–1.671	< 0.0001
Others	1.251	1.198–1.305	< 0.0001
**CVD comorbidity**			
No	1.000	Reference	–
Yes	2.254	2.179–2.330	< 0.0001
**Kt/V**			
< 0.8	2.382	2.210–2.736	< 0.0001
≥ 0.8 to < 1.0	1.706	1.612–1.801	< 0.0001
≥ 1.0 to < 1.2	1.295	1.238–1.355	< 0.0001
≥ 1.2 to < 1.4	1.000	Reference	–
≥ 1.4 to < 1.6	0.939	0.898–0.983	0.007
≥ 1.6 to < 1.8	0.812	0.767–0.859	< 0.0001
≥ 1.8 2.0	0.739	0.675–0.798	< 0.0001
≥ 2.0	0.762	0.682–0.851	< 0.0001
**β**_**2**_**- microglobulin (mg/L)**			
1 mg/L increase	1.031	1.029–1.034	< 0.0001
**C-reactive protein (mg/dL)**			
1 mg/dL increase	1.065	1.063–1.067	< 0.0001
**Hemoglobin (g/dL)**			
1 g/dL increase	0.767	0.758–0.775	< 0.0001
**Body mass index (kg/m**^**2**^**)**			
< 16	3.979	3.751–4.222	< 0.0001
≥ 16 to < 18	2.042	1.940–2.149	< 0.0001
≥ 18 to < 20	1.343	1.280–1.410	< 0.0001
≥ 20 to < 22	1.000	Reference	–
≥ 22 to < 24	0.814	0.767–0.863	< 0.0001
≥ 24 to < 26	0.669	0.618–0.724	< 0.0001
≥ 26 to < 28	0.677	0.608–0.753	< 0.0001
≥ 28	0.659	0.586–0.740	< 0.0001
**Serum albumin (g/dL)**			
< 3.0	7.301	7.016–7.597	< 0.0001
≥ 3.0 to < 3.5	2.542	2.451–2.636	< 0.0001
≥ 3.5 to < 4.0	1.000	Reference	–
≥ 4.0 to < 4.5	0.546	0.517–0.577	< 0.0001
≥ 4.5	0.475	0.391–0.576	< 0.0001
**nPCR (g/kg/day)**			
< 0.5	4.308	4.009–4.630	< 0.0001
≥ 0.5 to < 0.7	1.769	1.701–1.841	< 0.0001
≥ 0.7 to < 0.9	1.000	Reference	–
≥ 0.9 to < 1.1	0.698	0.671–0.727	< 0.0001
≥ 1.1 to < 1.3	0.671	0.627–0.716	< 0.0001
≥ 1.3	0.865	0.757–0.989	0.030
**SCI (mg/kg/day)**			
1 mg/kg/day increase	0.774	0.769–0.778	< 0.0001

*CVD* cardiovascular disease, *nPCR* normalized protein catabolic rate, *SCI* simplified creatinine index.

### Clinicodemographic characteristics by dialyzer type

Patients were subdivided into 5 groups by dialyzer type. Their clinical and demographic characteristics are shown in Table 3. In total, 81.3% of the patients underwent hemodialysis with type IV dialyzers, followed by types V (12.7%), III (4.1%), I (1.0%), and II (0.9%). Patients treated with a type I dialyzer were older and less likely to be male and had higher rates of comorbid CVD and presence of diabetes mellitus and a lower BMI. In contrast, the type V dialyzer group was younger and more likely to be male and had lower rates of CVD comorbidity and presence of diabetes mellitus and higher Kt/V, nPCR, and SCI.

**Table 3:** Clinicodemographic and laboratory values in 203,008 hemodialysis patients according to dialyzer type.

	I	II	III	IV	V	P value
n (%)	2087 (1.0)	1721 (0.9)	8349 (4.1)	165,082 (81.3)	25,769 (12.7)	
Age (years)	74.9 ± 10.7	70.8 ± 12.1	67.4 ± 12.4	65.6 ± 12.3	61.0 ± 12.2	< 0.0001
Sex (% female)	57.8	49.2	41.0	39.4	32.0	< 0.0001
Dialysis vintage (years)	5 [3–8]	5 [3–10]	6 [3–11]	7 [4–12]	8 [4–14]	< 0.0001
Presence of DM (%)	38.1	34.5	34.5	33.7	28.7	< 0.0001
**CVD comorbidity (%)**	34.9	33.7	27.3	25.4	19.9	< 0.0001
Coronary artery disease	8.2	9.9	7.5	7.4	6.2	
Ischemic stroke	23.2	22.1	17.6	15.2	11.1	
Hemorrhagic stroke	7.0	6.9	5.3	5.0	3.8	
Limb amputation	4.0	3.6	3.2	3.1	2.5	
Body mass index (kg/m^2^)	19.6 ± 3.4	20.2 ± 3.4	20.9 ± 3.5	21.1 ± 3.5	21.6 ± 3.5	< 0.0001
Hemoglobin (g/dL)	10.0 ± 1.4	10.1 ± 1.3	10.3 ± 1.3	10.4 ± 1.3	10.5 ± 1.2	< 0.0001
Serum albumin (g/dL)	3.5 ± 0.5	3.6 ± 0.5	3.6 ± 0.5	3.7 ± 0.4	3.7 ± 0.4	< 0.0001
Calcium (mg/dL)	8.8 ± 0.8	8.9 ± 0.8	9.0 ± 0.8	9.0 ± 0.8	9.1 ± 0.8	< 0.0001
Phosphate (mg/dL)	4.9 ± 1.5	5.2 ± 1.5	5.2 ± 1.5	5.3 ± 1.5	5.5 ± 1.5	< 0.0001
β_2_-microglobulin (mg/L)	31.1 ± 10.2	29.4 ± 8.8	28.2 ± 7.5	27.0 ± 6.8	27.2 ± 6.6	< 0.0001
C-reactive protein (mg/dL)	0.18 [0.06–0.75]	0.18 [0.07–0.56]	0.14 [0.06–0.43]	0.12 [0.05–0.40]	0.10 [0.05–0.30]	< 0.0001
Kt/V	1.26 ± 0.29	1.26 ± 0.27	1.38 ± 0.29	1.41 ± 0.29	1.43 ± 0.30	< 0.0001
nPCR (g/kg/day)	0.84 ± 0.20	0.83 ± 0.18	0.87 ± 0.19	0.88 ± 0.18	0.90 ± 0.17	< 0.0001
SCI (mg/kg/day)	18.4 ± 2.7	19.1 ± 2.9	20.3 ± 3.0	20.9 ± 3.0	22.2 ± 2.9	< 0.0001
Mortality rate (/person-years)	0.24	0.17	0.10	0.08	0.05	< 0.0001
**Mortality, n (%)**						0.002
All-cause	444 (21.3)	273 (15.9)	792 (9.5)	13,113 (7.9)	1278 (5.0)	
Cardiovascular-related	152 (7.3)	123 (7.2)	323 (3.9)	5518 (3.3)	559 (2.2)	
Infection-related	99 (4.7)	46 (2.7)	167 (2.0)	2342 (1.4)	206 (0.8)	
Cancer-related	33 (1.6)	29 (1.7)	79 (0.9)	1210 (0.7)	131 (0.5)	
Others	160 (7.7)	75 (4.4)	223 (2.7)	4043 (2.4)	382 (1.5)	

*CVD* cardiovascular disease, *DM* diabetes mellitus, *nPCR* normalized protein catabolic rate, *SCI* simplified creatinine index.

### All-cause mortality by dialyzer type

Compared with the type IV group (reference), the unadjusted HRs (95% confidence intervals [CIs]) for all-cause mortality in the type I, II, and III groups were 2.806 (2.555–3.081), 2.087 (1.856–2.348), and 1.206 (1.124–1.294), respectively (Supplementary Fig. 1). In contrast, only the type V group had a significantly lower HR—0.615 (0.581–0.651)—compared with the type IV group (reference). During the 195,828 person-years of follow-up, mortality rate was significantly and steadily lower in the groups with dialyzers providing higher β2MG clearance (Table 3).

**Figure 2:**
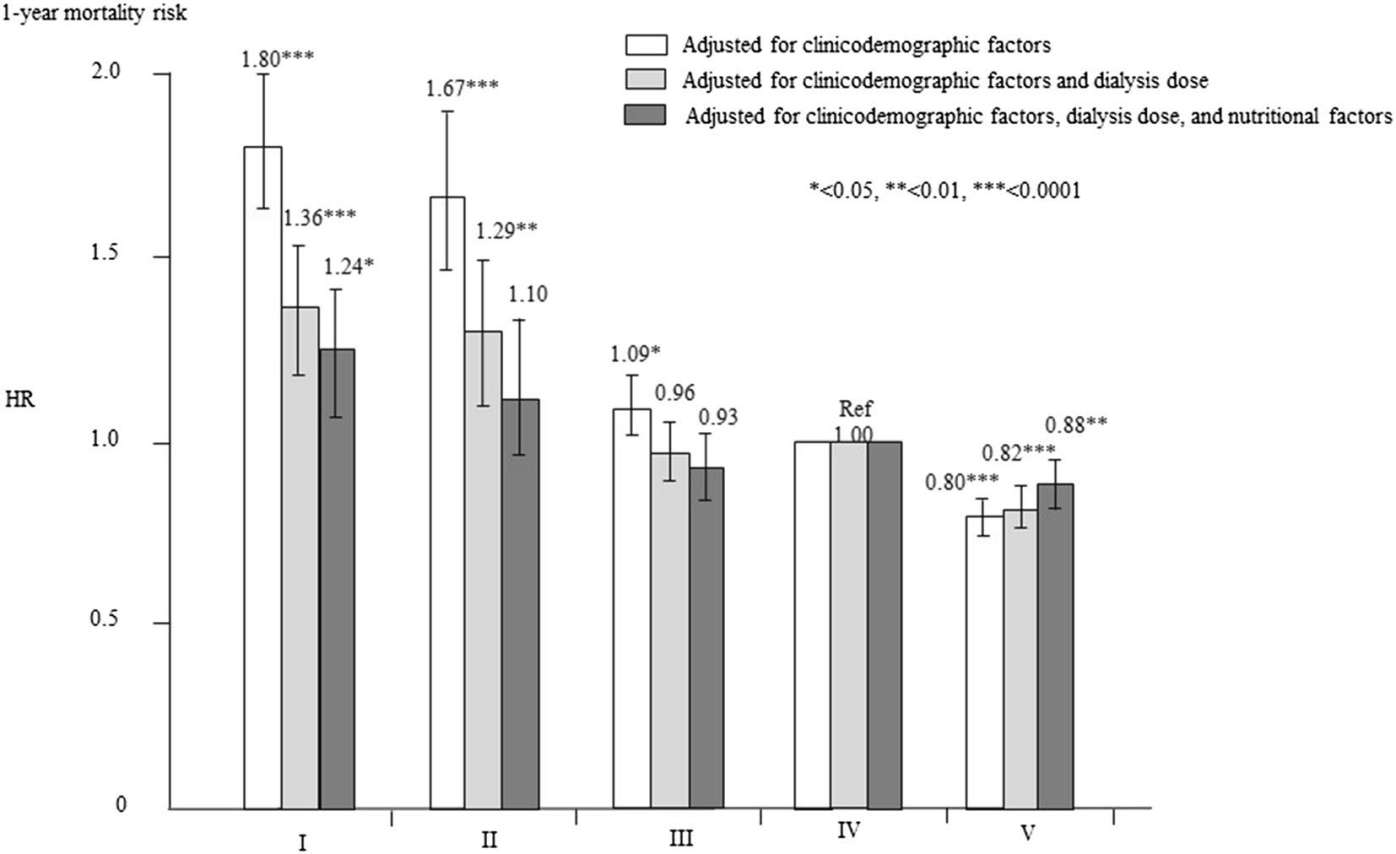
Hazard ratios of all-cause mortality for dialyzer type in 203,008 hemodialysis patients using a standard Cox proportional hazards regression. Bars with no fill are adjusted with clinicodemographic factors including age, sex, dialysis vintage, primary causes of end-stage kidney disease, and cardiovascular complication presence/absence. Gray-filled bars are adjusted with dialysis dose as assessed by Kt/V and β_2_-microglobulin levels in addition to clinicodemographic factors. Dark gray-filled bars are adjusted with clinicodemographic factors, dialysis dose, and nutrition- and inflammation-related factors, including body mass index, hemoglobin, C-reactive protein, and serum albumin levels, normalized protein catabolic rate, and simplified creatinine index. *P < 0.05, **P < 0.01, and ***P < 0.0001 versus the type IV dialyzer group. Error bars correspond to 95% confidence intervals.

The adjusted HRs for all-cause mortality of each group are shown in Fig. 2. After adjustment for clinicodemographic factors and compared with the type IV group (reference), the HRs (95% CIs) for all-cause mortality in the type I, II, and III groups were 1.803 (1.629–1.995), 1.668 (1.463–1.901), and 1.086 (1.006–1.172), respectively. The type V group had a significantly lower HR—0.803 (0.755–0.854)—compared with the type IV group (Supplementary Table 2).

After adjustment for dialysis dose and β2MG, as well as clinicodemographic factors, the HRs in the type I, II, and III groups compared with the type IV group (reference) were 1.364 (1.216–1.529), 1.286 (1.097–1.508), and 0.958 (0.875–1.051), respectively. The type III group showed no significant difference from the type IV group, whereas the type V group had a significantly lower HR of 0.821 (0.761–0.881).

Finally, after adjustment for nutrition- and inflammation-related factors, in addition to clinicodemographic factors and dialysis dose, the HRs in the type II and III groups were not significantly different compared with the type IV group. The type I group had a significant higher HR (95% CI) of 1.244 (1.046–1.442) compared with the type IV group but the lower HR in the type V group persisted (0.878 [0.811–0.951], P = 0.001).

### Propensity scored-matched analysis

**Figure 3:**
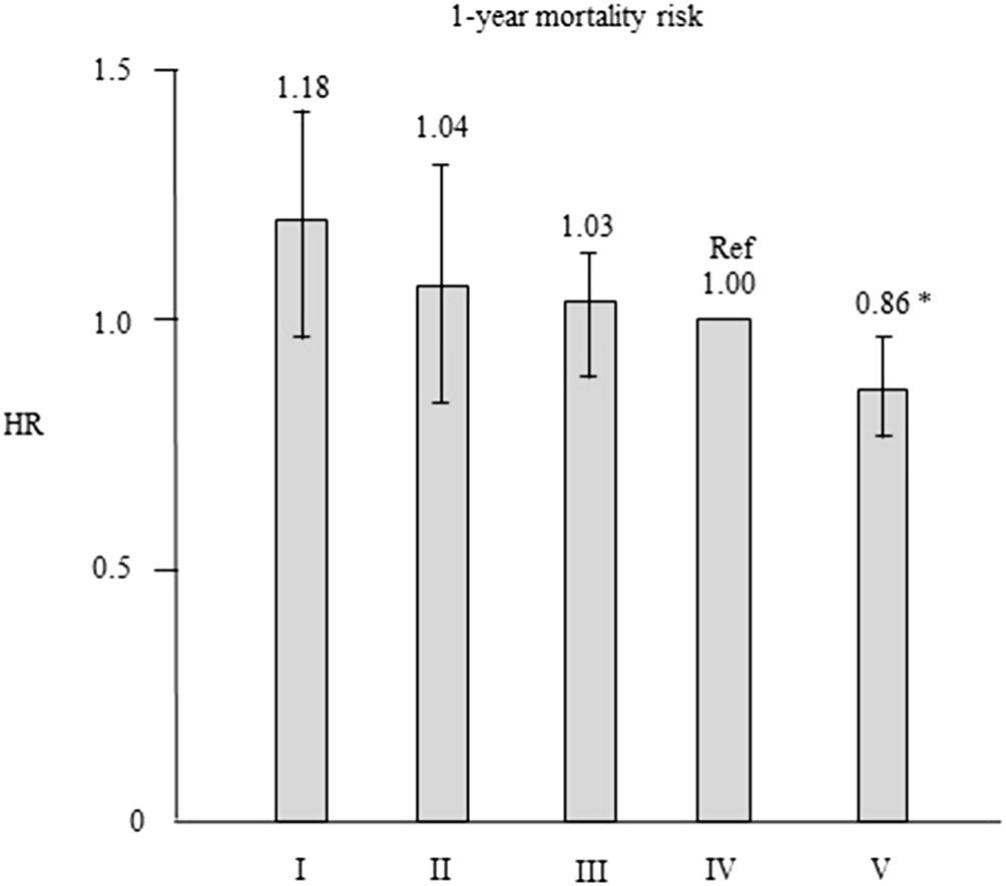
Hazard ratios of all-cause mortality for the 4 dialyzer groups versus the type IV dialyzer group after propensity score matching using a Cox proportional hazards regression. *P < 0.05 versus the type IV dialyzer group. Error bars correspond to 95% confidence intervals.

Patients’ characteristics and clinical data at baseline in the type IV group and each corresponding group after propensity score matching are shown in Table 4. None of the variables were significantly different among the groups. As can be seen in Fig. 3, the HRs in the type I, II, and III groups were not significantly different compared with the type IV group. The HR (95% CI) in the type V group (0.862 [0.770–0.965], P = 0.010) was significantly lower than that in the type IV group. We employed the IPTW method to perform a sensitivity analysis by incorporating the values estimated by the propensity scores. Cox’s proportional hazard models adjusted by IPTW gave results similar to those of the propensity scored-matched analysis (Supplementary Fig. 2).

**Table 4:** Baseline characteristics after propensity score matching between type IV and other types of dialyzers.

	Matched			Matched			Matched			Matched		
	I	IV	P value	II	IV	P value	III	IV	P value	V	IV	P value
n (%)	1104	1104	–	796	796	–	3867	3867	–	13,011	13,011	–
Age (years)	75.2 ± 10.6	75.6 ± 10.2	0.385	71.2 ± 11.6	71.2 ± 11.4	0.945	67.7 ± 12.2	67.8 ± 11.8	0.740	60.9 ± 12.1	60.9 ± 12.5	0.992
Sex (% female)	59.8	59.7	0.965	51.2	51.6	0.871	40.2	41.1	0.392	49.2	57.8	0.424
Dialysis vintage (years)	5 [3–8]	5 [3–8]	0.725	4.5 [3–9]	5 [3–9]	0.461	6 [3–10]	6 [3–10]	0.554	8 [4–14]	8 [4–14]	0.704
Presence of DM (%)	36.4	37.8	0.481	37.8	37.1	0.779	36.6	35.3	0.209	28.4	28.7	0.546
CVD comorbidity (%)	34.1	33.4	0.718	31.7	31.3	0.861	27.6	27.6	0.979	20.3	20.7	0.416
BMI (kg/m^2^)	19.6 ± 3.3	19.6 ± 3.2	0.801	20.4 ± 3.3	20.6 ± 3.4	0.460	20.9 ± 3.5	20.9 ± 3.5	0.834	21.6 ± 3.5	21.6 ± 3.5	0.854
Hemoglobin (g/dL)	10.1 ± 1.4	10.1 ± 1.3	0.532	10.2 ± 1.2	10.2 ± 1.2	0.912	10.3 ± 1.3	10.3 ± 1.3	0.108	10.6 ± 1.2	10.6 ± 1.2	0.819
Serum albumin (g/dL)	3.5 ± 0.5	3.5 ± 0.5	0.160	3.6 ± 0.4	3.6 ± 0.4	0.855	3.7 ± 0.4	3.7 ± 0.4	0.596	3.8 ± 0.4	3.8 ± 0.4	0.616
Calcium (mg/dL)	8.8 ± 0.8	8.8 ± 0.9	0.582	8.8 ± 0.8	8.9 ± 0.8	0.295	9.0 ± 0.8	9.0 ± 0.8	0.471	9.0 ± 0.8	9.0 ± 0.8	0.876
Phosphate (mg/dL)	4.9 ± 1.4	4.9 ± 1.4	0.143	5.1 ± 1.4	5.1 ± 1.3	0.721	5.2 ± 1.4	5.2 ± 1.4	0.824	5.4 ± 1.4	5.4 ± 1.4	0.901
β2MG (mg/L)	30.9 ± 9.8	30.9 ± 9.9	0.188	29.0 ± 9.0	29.1 ± 8.8	0.750	27.8 ± 7.8	27.8 ± 7.3	0.899	27.1 ± 6.5	27.1 ± 6.6	0.412
C-reactive protein (mg/dL)	0.16 [0.06–0.60]	0.20 [0.08–0.61]	0.241	0.12 [0.05–0.45]	0.16 [0.07–0.52]	0.805	0.13 [0.06–0.40]	0.13 [0.06–0.40]	0.261	0.10 [0.05–0.30]	0.10 [0.05–0.30]	0.885
Kt/V	1.27 ± 0.28	1.27 ± 0.29	0.945	1.28 ± 0.27	1.28 ± 0.28	0.703	1.37 ± 0.28	1.38 ± 0.29	0.102	1.45 ± 0.30	1.44 ± 0.29	0.119
nPCR (g/kg/day)	0.83 ± 0.18	0.83 ± 0.18	0.645	0.83 ± 0.17	0.83 ± 0.17	0.831	0.85 ± 0.17	0.86 ± 0.17	0.679	0.90 ± 0.17	0.90 ± 0.17	0.311
SCI (mg/kg/day)	18.4 ± 2.7	18.3 ± 2.6	0.241	19.0 ± 2.9	18.9 ± 2.7	0.317	20.3 ± 3.0	20.6 ± 3.0	0.936	22.2 ± 2.9	22.2 ± 2.9	0.696

*β2MG* β_2_-microglobulin, *BMI* body mass index, *CVD* cardiovascular disease, *DM* diabetes mellitus, *nPCR* normalized protein catabolic rate, *SCI* simplified creatinine index.

## Discussion

Here, we determined the predictors of 1-year mortality in patients on hemodialysis, which included clinicodemographic factors such as male sex, older age, dialysis vintage, end-stage kidney disease cause, and comorbid CVD, as previously reported^16,17^. Additional predictors were dialysis-related factors such as Kt/V and β2MG and nutrition- and inflammation-related factors. Moreover, we compared mortality rates among 5 types of dialyzers with adjustment for multiple predictive factors. After full adjustment for these factors, the HR for the type V group was significantly lower than that for the type IV (reference) group. Finally, analysis of propensity score-matched cohorts confirmed that the HR for the type V group was significantly lower than that for the type IV group. In particular, cardiovascular mortality, which is the main cause of death in hemodialysis patients, was significantly lower in type V group compared with the other groups. Our results are the first to indicate that the mortality risk of patients on hemodialysis might be influenced by the type of dialyzer used and that HPM or super high-flux dialyzers might improve the outcomes of hemodialysis patients.

Low- and high-flux hemodialysis were compared in 2 large randomized controlled trials, the Membrane Permeability Outcome (MPO) study and the Hemodialysis (HEMO) study^18,19^. The HEMO study failed to identify a better outcome between the high-flux and low-flux groups. However, a significantly better survival was found with high-flux hemodialysis in a subgroup analysis of patients who had received hemodialysis for longer than 3.7 years, with a relative risk reduction of 32%^20^. Moreover, pre-dialysis serum β2MG levels but not β2MG dialyzer clearance were associated with all-cause mortality^21^. The MPO study included 738 patients on hemodialysis. The results indicated no significant effect of high-flux hemodialysis on mortality in the overall population. However, subgroup analysis of patients with serum albumin levels ≤ 4 g/dL revealed significantly improved survival in the high-flux group, with a relative risk reduction of 37%^19^. In addition, subgroup analysis also identified significantly longer survival of patients with diabetes in the high-flux group than in the low-flux group, and a relative risk reduction of 38%^19^. Considering these findings, European Renal Best Practice guidelines advise the use of high-flux and highly biocompatible dialyzers in high-risk patients, whereas Kidney Disease Outcomes Quality Initiative guidelines discourage the use of cellulose dialyzers with low biocompatibility^2,3^. In the HEMO study, high-flux dialyzers were defined as those with an ultrafiltration coefficient ≥ 14 mL/h/mmHg and a mean β2MG clearance > 20 mL/min, which resulted in an actual β2MG clearance of 33.8 ± 11.4 mL/min in the high-flux group^18^. In the MPO study, high-flux dialyzers were defined as those with an ultrafiltration coefficient ≥ 20 mL/h/mmHg and a sieving coefficient for β2MG > 0.6^19^. On the other hand, 94% of the patients were treated with type IV and V dialyzers, which were defined as β2MG clearance ≥ 50 mL/min. Therefore, many of the patients in the present study were treated with more efficient dialyzers that surpass the conventional high-flux dialyzers used in previous studies.

In Japan, kidney transplantations are performed in selected patients, which has led to a year-on-year increase in the number of hemodialysis patients on long-term dialysis. The present study included elderly patients and patients with a longer dialysis vintage. CVD and malnutrition comorbidities are common in patients receiving long-term dialysis and are associated with physical disability and morbidity. Therefore, to ameliorate the comorbidities of long-term dialysis therapy and improve outcomes, HPM dialyzers became the subject of major research efforts in Japan, which managed to improve their properties. The principal goal of HPM dialyzers is to remove uremic toxins with molecular weights of 10–30 kDa and they are characterized as having high biocompatibility, high hydraulic permeability, and high solute permeability, particularly for middle-molecular-weight molecules^5^. JSDT recommendations advise the use of HPM dialyzers in patients on hemodialysis due to their ability to improve prognosis and decrease dialysis-related complications^4^. Although the efficacy of medium cut-off (MCO) membrane dialyzers is focused on hemodiafiltration treatment, the characteristics of the type IV and V dialyzers used here are similar to those of MCO membrane dialyzers^22,23^. Type IV and V dialyzers are composed of synthetic membranes and are classified as HPM dialyzers or so-called “super high-flux” dialyzers. The present work is the first to determine which of the 5 types of dialyzers achieves good prognosis and is not simply a comparison of high- versus low-flux dialyzers.

The selection of highly biocompatible dialyzers and purified dialysate is crucial to minimize inflammatory responses among hemodialysis patients. The use of dialyzers that rapidly trigger the complement system, leukocytosis, and inflammatory response is discouraged in several guidelines^2–4^. Although a 2005 meta-analysis failed to identify the superiority of synthetic polymer membranes, a more recent meta-analysis revealed an approximate 15% reduction in cardiovascular mortality in patients requiring hemodialysis with high-flux dialyzers^24,25^. Furthermore, the mortality rate is lower in patients with serum β2MG levels of 27.5–34.0 mg/L, and albumin-bound uremic toxins and low-molecular-weight proteins, such as α1-microglobulin (α1MG), are being targeted for removal to improve prognosis in patients on hemodialysis^21,26^. Furthermore, it has been reported the removal of albumin that is bound to biologically active uremic substances and/or removal of the oxidized form of albumin that has lost its antioxidant activity might be beneficial in dialysis patients^26,27^. Accordingly, some degree of albumin leakage would be useful for removing biologically active uremic toxins bound to albumin and oxidized albumin and for facilitating the synthesis of new albumin with antioxidant activity. Albumin leakage of many type V dialyzers does not exceed 3 g^28^. Type V dialyzers are characterized by higher β2MG clearance and higher biocompatibility. In addition, a certain degree of albumin leakage due to type V dialyzers may contribute to the removal of middle molecular weight uremic proteins. However, some type V dialyzers have approximately 8 g albumin leakage, which would lead to reduced serum albumin levels and dyslipidemia^29^. Albumin leakage is often suggested as a disadvantage of albumin-leaky hemodialysis, but the patients in the type V dialyzer group had the highest serum albumin levels among the dialyzer groups. Therefore, large amounts of albumin leakage, which would lead to hypoalbuminemia, did not occur in the present study. However, longer-term use of membranes with albumin leakage, such as with type V dialyzers, may still pose a risk of decreased serum albumin levels or malnutrition, and longitudinal studies are needed to evaluate this. Additional investigations are needed to determine the risks and benefits of albumin leakage with dialyzers and the amounts of albumin leakage considered acceptable.

Some limitations of our work should be mentioned. First, given the nature of the annual survey and observational cohort, the numbers of patients differed among the 5 types of dialyzers. Laboratory parameters were evaluated at only one time point (i.e., at baseline) but their values may have changed over the study period. Second, the present study could not investigate center effects. Mortality might differ among the facilities due to differences in patient populations and in facility practices such as anemia control. Also, selection bias might be present because the type I dialyzer group had lower BMI, nPCR, and SCI and a higher rate of CVD comorbidity. However, we confirmed the nonsuperiority of type I dialyzers and superiority of type V dialyzers after propensity score-matched analysis. Third, we could not determine quality-adjusted life years because this survey did not collect data on quality of life. Also, in Japan, the costs of the dialyzers differ according to their type and membrane area. Type I of < 1.5 m^2^ is cheapest (13.8 USD) and type V of ≥ 1.5 m^2^ is the most expensive (20.2 USD), so a cost effectiveness analysis might be needed in the future. Fourth, the present study included patients with dialysis vintage of several years, which means they were a selected group of survivors. Cardiovascular disease is the leading cause of death among Japanese dialysis patients, but infection-related death is the leading cause of death among incident dialysis patients^30^. Therefore, further investigation is needed to clarify whether type V dialyzers improve prognosis even in incident patients. Finally, patients treated with hemodiafiltration were excluded to eliminate a modality bias and due to the small number of these patients in Japan in 2008^9^. However, hemodiafiltration is considered a more efficient modality for the use of high-flux dialyzers because it can achieve a higher clearance of small substances and middle-molecular-weight substances such as β2MG and α1MG compared with conventional high-flux hemodialysis^31–33^. Therefore, further research is required to elucidate the differences among treatment modalities.

To conclude, using a nationwide cohort study of patients on hemodialysis in Japan, we determined that treatment with type V dialyzers was associated with significantly improved 1-year mortality compared with type IV dialyzers. Our findings provide evidence that treatment with HPM or super high-flux dialyzers has beneficial effects on hemodialysis patients and highlight the need to consider the use of these dialyzers in hemodialysis patients.

## Supplementary Information

Supplementary Information 1.

Supplementary Information 2.

Supplementary Information 3.

Supplementary Information 4.

Supplementary Information 5.
