# Supplementary material for: High-performance dialyzers and mortality in maintenance hemodialysis patients
Source: Sci Rep. 2021 Jun 10;11:12272. doi: 10.1038/s41598-021-91751-w (PMC8192518; doi:10.1038/s41598-021-91751-w)
Supplement: Supplementary file 1 — Supplementary Information 1. [file 41598_2021_91751_MOESM1_ESM.pdf]

Supplementary Table 1. Proportion of each categorical variable in 203,008 maintenance hemodialysis patients

Variable	Proportion (%)
Dialysis duration, years	
> 1 to < 5	40.7
≥ 5 to < 10	29.1
≥ 10 to < 15	14.4
≥ 15 to < 25	7.4
≥ 25 to < 30	6.8
≥ 30	1.6
Body mass index, kg/m^2	
< 16	4.1
≥ 16 to < 18	12.6
≥ 18 to < 20	23.2
≥ 20 to < 22	24.5
≥ 22 to < 24	17.7
≥ 24 to < 26	9.5
≥ 26 to < 28	4.5
≥ 28	3.9
Albumin, g/dL	
< 3.0	4.6
≥ 3.0 to < 3.5	18.1
≥ 3.5 to < 4.0	50.7
≥ 4.0 to < 4.5	24.7
≥ 4.5	1.9

Kt/V	
< 0.8	1.1
≥ 0.8 to < 1.0	4.9
≥ 1.0 to < 1.2	16.4
≥ 1.2 to < 1.4	28.6
≥ 1.4 to < 1.6	25.3
≥ 1.6 to < 1.8	14.4
≥ 1.8 to < 2.0	6.1
≥ 2	3.2
nPCR, g/kg/day	
< 0.5	1.0
≥ 0.5 to < 0.7	12.8
≥ 0.7 to < 0.9	40.6
≥ 0.9 to < 1.1	34.0
≥ 1.1 to < 1.3	9.9
≥ 1.3	1.7

nPCR, normalized protein catabolic rate.
