# Supplementary material for: High-performance dialyzers and mortality in maintenance hemodialysis patients
Source: Sci Rep. 2021 Jun 10;11:12272. doi: 10.1038/s41598-021-91751-w (PMC8192518; doi:10.1038/s41598-021-91751-w)
Supplement: Supplementary file 2 — Supplementary Information 2. [file 41598_2021_91751_MOESM2_ESM.pdf]

Supplementary Table 2. Hazard ratios (95% CIs) of all-cause mortality between the five types of dialyzers in 203,008 maintenance hemodialysis patients using a standard Cox proportional hazards regression

Dialyzers	Unadjusted			Adjusted for clinicodemographic factors [a]			Adjusted for clinicodemographic factors and dialysis dose [b]			Adjusted for clinicodemographic factors, dialysis dose, and nutrition- and inflammation-related factors [c]		
	HR	95% CI	P value	HR	95% CI	P value	HR	95% CI	P value	HR	95% CI	P value
I	2.81	(2.56–3.08)	<0.0001	1.80	(1.63–1.99)	<0.0001	1.36	(1.21–1.53)	<0.0001	1.24	(1.05–1.44)	0.014
II	2.09	(1.86–2.35)	<0.0001	1.67	(1.46–1.90)	<0.0001	1.29	(1.10–1.51)	0.0028	1.14	(0.96–1.35)	0.126
III	1.21	(1.12–1.29)	<0.0001	1.09	(1.01–1.17)	0.037	0.96	(0.88–1.05)	0.363	0.93	(0.84–1.03)	0.187
IV	1.00	Reference	Reference	1.00	Reference	Reference	1.00	Reference	Reference	1.00	Reference	Reference
V	0.62	(0.58–0.65)	<0.0001	0.80	(0.75–0.85)	<0.0001	0.82	(0.76–0.88)	<0.0001	0.88	(0.81–0.95)	0.0014

[a], adjusted for age, sex, dialysis duration, presence or absence of diabetes, and cardiovascular disease; [b], adjusted for clinicodemographic factors, Kt/V, and β_2-microglobulin; [c], adjusted for clinicodemographic factors, Kt/V, β_2-microglobulin, C-reactive protein, hemoglobin, normalized protein catabolic rate, serum albumin, body mass index, simplified creatinine index.
