# Supplementary material for: High-performance dialyzers and mortality in maintenance hemodialysis patients
Source: Sci Rep. 2021 Jun 10;11:12272. doi: 10.1038/s41598-021-91751-w (PMC8192518; doi:10.1038/s41598-021-91751-w)
Supplement: Supplementary file 3 — Supplementary Information 3. [file 41598_2021_91751_MOESM3_ESM.docx]

**Supplementary Figure legends**

**Supplementary Figure 1.** Hazard ratios of unadjusted all-cause mortality for dialyzer type in 203,008 hemodialysis patients using a standard Cox proportional hazards regression.

**Supplementary Figure 2.** Hazard ratios of all-cause mortality for dialyzer type using a standard Cox proportional hazards regression with inverse probability of treatment weighting.
