# Supplementary figures and images for: High-performance dialyzers and mortality in maintenance hemodialysis patients
Source: Sci Rep. 2021 Jun 10;11:12272. doi: 10.1038/s41598-021-91751-w (PMC8192518; doi:10.1038/s41598-021-91751-w)

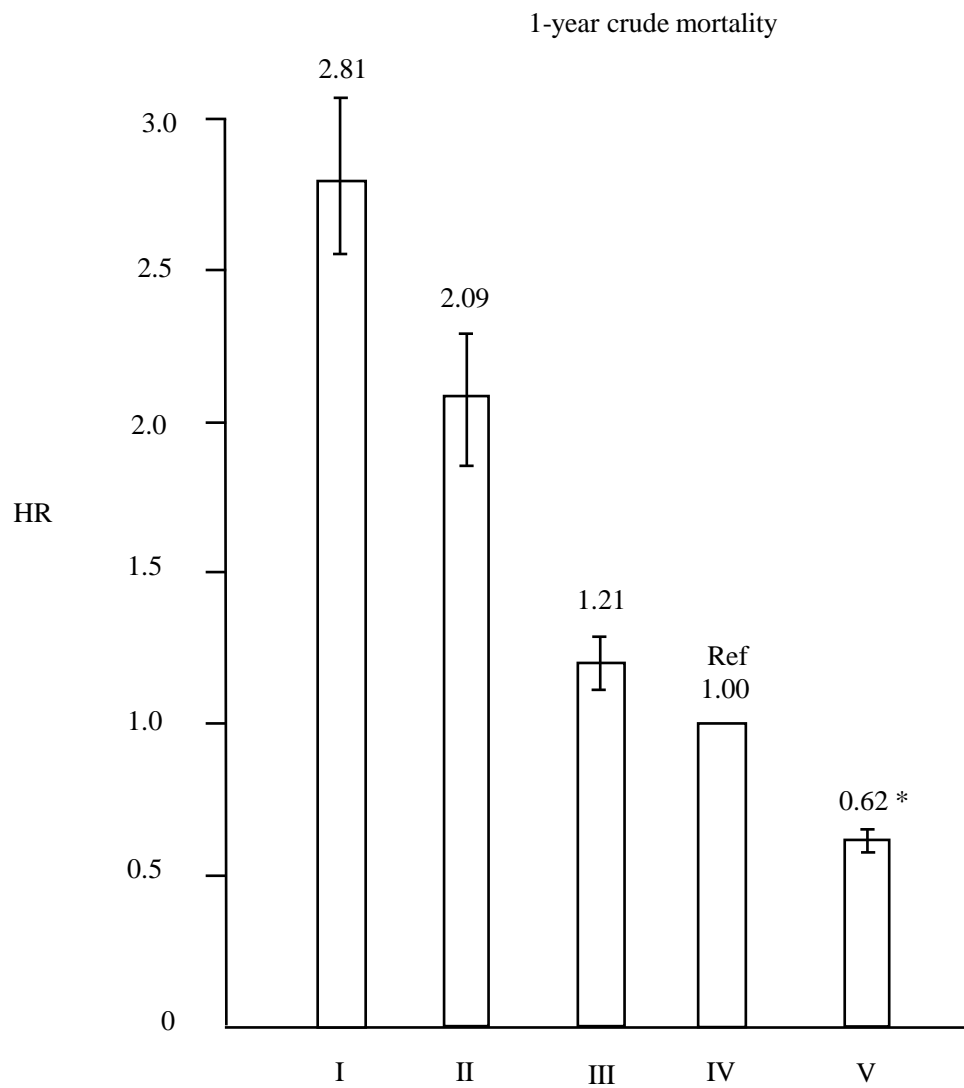

Supplementary Figure 1

Supplement: Supplementary file 4 — Supplementary Information 4. [file 41598_2021_91751_MOESM4_ESM.pdf]

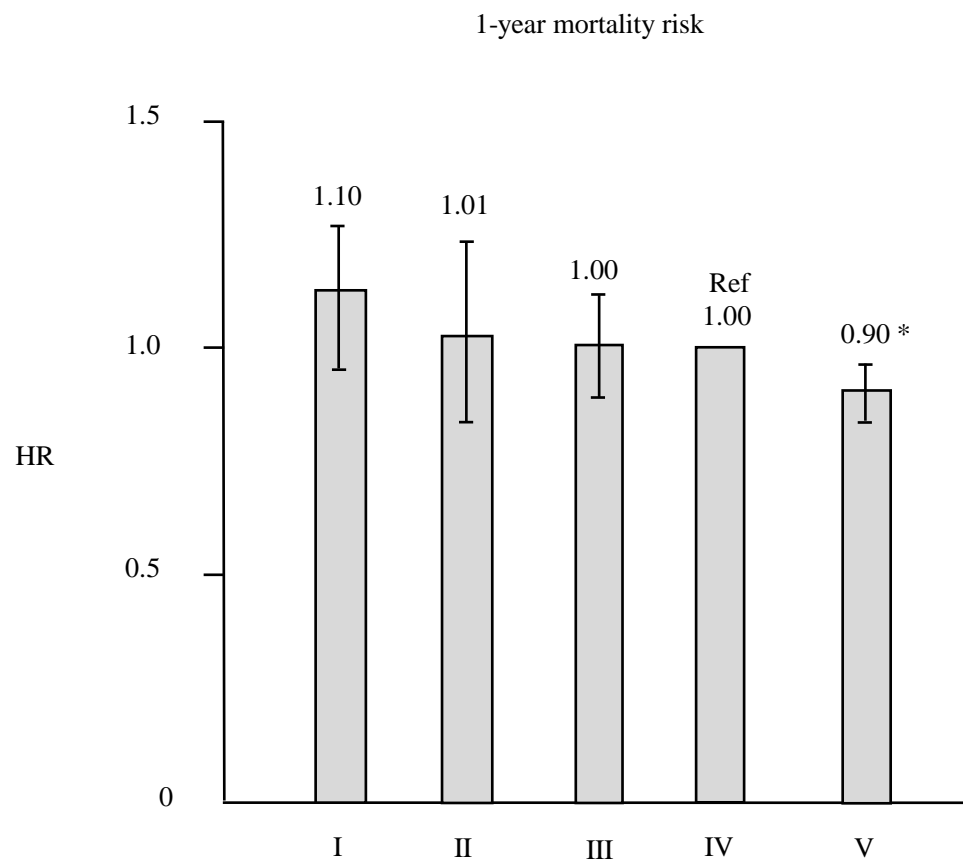

Supplement: Supplementary file 5 — Supplementary Information 5. [file 41598_2021_91751_MOESM5_ESM.pdf]
